# Supplementary material for: Automatic airway segmentation from computed tomography using robust and efficient 3-D convolutional neural networks
Source: Sci Rep. 2021 Aug 6;11:16001. doi: 10.1038/s41598-021-95364-1 (PMC8346579; doi:10.1038/s41598-021-95364-1)
Supplement: Supplementary file 1 — Supplementary Information 1. [file 41598_2021_95364_MOESM1_ESM.pdf]

Supplementary Information

Automatic airway segmentation from Computed Tomography using robust and efficient 3-D convolutional neural networks

Antonio Garcia-Uceda[1,2,*], Raghavendra Selvan[3,4], Zaigham Saghir[5], Harm A.W.M. Tiddens[1,2], Marleen de Bruijne[1,3,*]

[1] Department of Radiology and Nuclear Medicine, Erasmus MC, 3015 CE, Rotterdam, The Netherlands
[2] Department of Pediatric Pulmonology and Allergology, Erasmus MC-Sophia Children Hospital, 3015 CE, Rotterdam, The Netherlands
[3] Department of Computer Science, University of Copenhagen, 2100, Copenhagen, Denmark
[4] Department of Neuroscience, University of Copenhagen, 2200, Copenhagen, Denmark
[5] Department of Medicine, Section of Pulmonary Medicine, Herlev-Gentofte Hospital, Copenhagen University Hospital, 2900, Hellerup, Denmark
[*] a.garciauceda@erasmusmc.nl
[*] marleen.debruijne@erasmusmc.nl

Appendix

Learning curves of the proposed method

We computed the learning curve for the proposed U-Net-based-method trained on both CF-CT and DLCST data together. To do this, we trained several models with different sizes of the training data. The maximum training size has half of the CT scans from the CF-CT and DLCST data (28 scans in total), which is the same data we used to train the model evaluated on the EXACT'09 data in this paper. We keep the remaining 28 scans for testing the trained models. For each training set used, the ratio between CF-CT and DLCST scans is the same as in the full dataset. We did three experiments for each training size, with randomly assigned training images (except for the largest run with 28 scans). To compute the airway predictions on the test data, we did not extract the largest connected component from the thresholded output of the U-Net, as we did for the other experiments in this paper. This is to account for the full prediction of the U-Net in assessing the method accuracy for all training sizes. To compare the results for different training sizes, we applied the paired, two-sided Student's T-test on the average of the measures from the three experiments for a given size, and consider that a p-value lower than 0.01 indicates a significant difference. We show in Fig. 1 the computed learning curves, with the different performance metrics obtained for each run and training set size. The measures of tree length detected increase progressively with the training size. The difference between the scores with sizes of 18 and 28 images is still significant ($p < 0.001$), and adding more training images could still improve slightly the results. For the measures of centerline leakage and Dice coefficient, they are more similar between sizes of 9 and 18 images ($p = 0.35$ and $p = 0.26$, respectively) and between sizes of 18 and 28 images ($p = 0.99$ and $p = 0.019$, respectively).

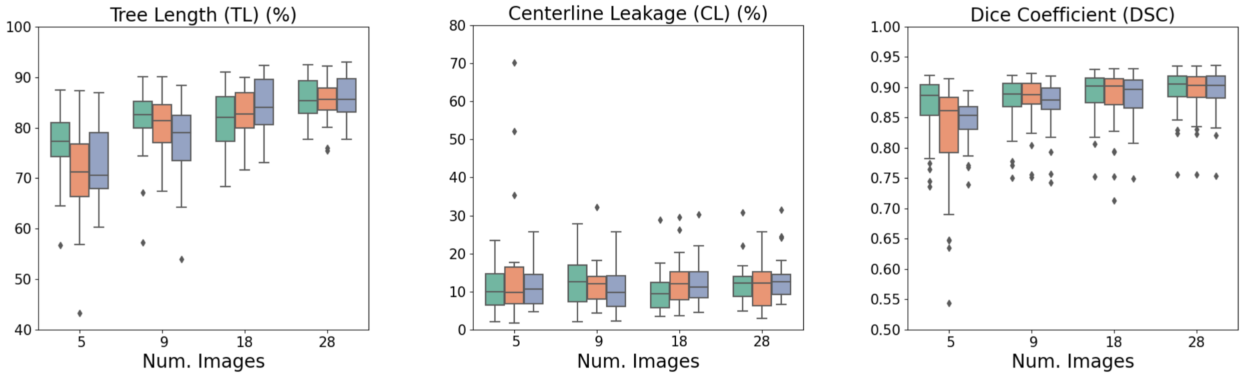

Figure 1: Learning curve for our U-Net-based-method trained on both CF-CT and DLCST data together. Boxplots showing i) tree length detected, ii) centerline leakage and iii) Dice coefficient on both CF-CT and DLCST data together, for each experiment and training size. For each boxplot, the box shows the quartiles of the data (defined by the median, 25% and 75% percentiles), the whiskers extend to include the data within 1.5 times the interquartile range from the box limits, and the markers show the data outliers.

Results of the proposed method grouped by the presence of lung disease

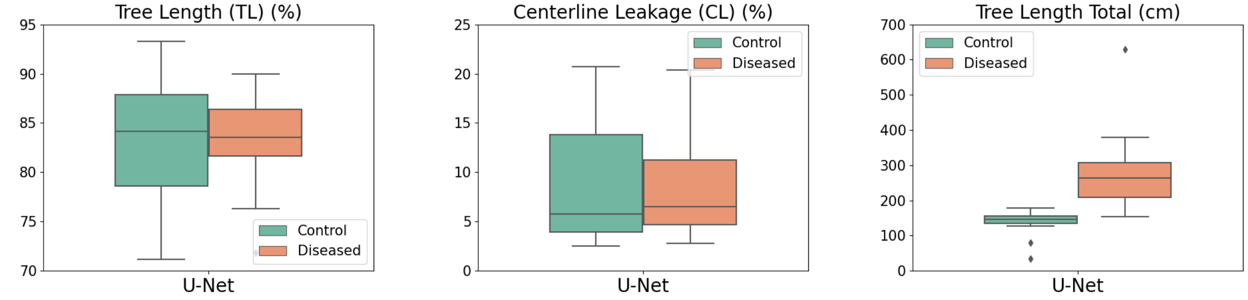

Figure 2: Boxplots showing i) tree length detected, ii) centerline leakage and iii) total tree length on the CF-CT data, grouped by the presence of CF disease in the CT scans, for the results obtained with our U-Net-based method. For each boxplot, the box shows the quartiles of the data (defined by the median, 25% and 75% percentiles), the whiskers extend to include the data within 1.5 times the interquartile range from the box limits, and the markers show the data outliers.

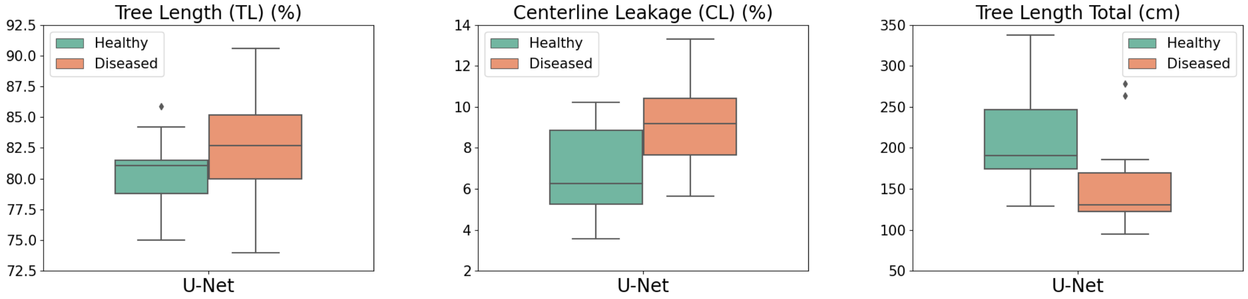

Figure 3: Boxplots showing i) tree length detected, ii) centerline leakage and iii) total tree length on the DLCST data, grouped by the presence of COPD disease in the CT scans, for the results obtained with our U-Net-based method. For each boxplot, the box shows the quartiles of the data (defined by the median, 25% and 75% percentiles), the whiskers extend to include the data within 1.5 times the interquartile range from the box limits, and the markers show the data outliers.

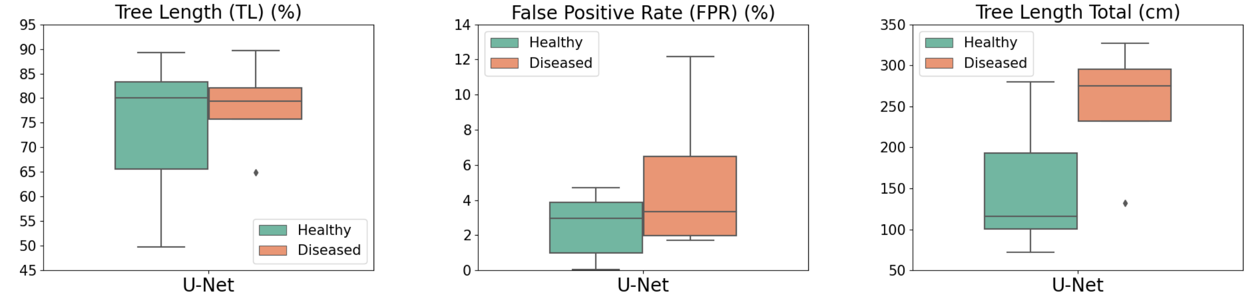

Figure 4: Boxplots showing i) tree length detected, ii) false positive rate and iii) total tree length on the EXACT'09 test data, grouped by the presence of bronchiectasis disease in the CT scans, for the results obtained with our U-Net-based method. The healthy group contains 7 scans without any reported anomalies on the CT scan, while the diseased group contains 4 scans with reported bronchiectasis [13]. For each boxplot, the box shows the quartiles of the data (defined by the median, 25% and 75% percentiles), the whiskers extend to include the data within 1.5 times the interquartile range from the box limits, and the markers show the data outliers.

Implementation details of the nnU-Net method and our experiments for airway segmentation

The nnU-Net method proposed by Isensee et al. [46] is a general segmentation framework designed for biomedical segmentation tasks. We applied the method for airway segmentation from chest CTs. We used the implementation in `https://github.com/MIC-DKFZ/nnUNet`.

For our experiment, we used the so-called "full3d_UNet" in the nnU-Net framework, which is the most similar to the U-Net in our method. This U-Net has 5 levels of resolution, where each level in the downsampling / upsampling path has two 3×3×3 convolutional layers and a 2×2×2 pooling / deconvolution layer, respectively. Each convolution operation is followed by an instance normalisation layer and a leaky rectified linear (leaky-ReLU) activation. The number of features in the top resolution level is 32, and after each pooling / deconvolution layer the number of feature channels is halved / doubled, respectively. The network uses deep supervision at all levels of the U-Net. In this, the output of every last convolutional layer at every resolution level is concatenated, after being resampled to the original resolution. Then, the final layer is a 1x1x1 convolutional layer followed by a sigmoid activation function.

To train the network, we used the same 28 CT scans and ground truth segmentations from the CF-CT and DLCST data as we used to train our method. The nnU-Net method uses as training loss function a combination of the i) binary cross entropy and ii) soft Dice losses. We could not modify the loss computation in the nnU-Net to consider only voxels within the lung regions, as we did for our method in equation (1). Instead, we masked the ground truth segmentations to the mask of the lung fields, to remove the trachea and part of the main bronchi. The nnU-Net method uses the SGD optimizer with an adaptable learning rate, starting with a value of 10^{-2}. We trained the model for a sufficiently large number of epochs, 600, until the training and validation losses are clearly stabilized. We then retrieved the model with the overall minimum validation loss for testing, denoted by "model_best" in the nnU-Net framework. Training time was approximately 2-3 days on a GPU GeForce RTX 2080 Ti. Test time inference takes between 5-15 min per scan, including pre-processing.

The nnU-Net applies some pre-processing operations to the scans and ground truth in the training dataset. First, the images are cropped to the region of non-zero values in the ground truth airway masks. Then, the images are resampled to a fixed resolution equal to the median over the training dataset, using 3rd order spline interpolation for the CTs and nearest neighbour interpolation for the ground truth masks. During training, the nnU-Net extracts random patches from the scans and ground truth, with a total of 1 patch per image and per epoch. Then, random rigid transformations are applied for data augmentation, including i) flipping in the three directions, ii) random small 3D rotations, and iii) random scaling.

At inference time, the nnU-Net applies the same pre-processing operations to the test scans as those applied on the training data used for the tested model. The images are resampled to the same fixed resolution used for the training data, i.e. the median over these data. Input patches to the network are extracted from the scans in a sliding-window fashion, with an overlap of roughly 50% between the patches in each direction, and then are processed through the trained model. The output predicted patches containing airway probability maps are aggregated and the full-size output is reconstructed. Then, thresholding is used to obtain the airway binary segmentation, and the pre-processing steps are reversed to recover the original image resolution. Finally, we merge this segmentation with a mask for the trachea, main bronchi, and the first 5 voxels of the next branches to obtain the full airway tree, which is easily computed by a region growing method [9].
